# Allele detection using *k*-mer-based sequencing error profiles

**DOI:** 10.1093/bioadv/vbad149

**Published:** 2023-10-20

**Authors:** Hufsah Ashraf, Jana Ebler, Tobias Marschall

**Affiliations:** Institute for Medical Biometry and Bioinformatics, Medical Faculty, Heinrich Heine University, 40225 Düsseldorf, Germany; Center for Digital Medicine, Heinrich Heine University, 40225 Düsseldorf, Germany; Institute for Medical Biometry and Bioinformatics, Medical Faculty, Heinrich Heine University, 40225 Düsseldorf, Germany; Center for Digital Medicine, Heinrich Heine University, 40225 Düsseldorf, Germany; Institute for Medical Biometry and Bioinformatics, Medical Faculty, Heinrich Heine University, 40225 Düsseldorf, Germany; Center for Digital Medicine, Heinrich Heine University, 40225 Düsseldorf, Germany

## Abstract

**Motivation:**

For genotype and haplotype inference, typically, sequencing reads aligned to a reference genome are used. The alignments identify the genomic origin of the reads and help to infer the absence or presence of sequence variants in the genome. Since long sequencing reads often come with high rates of systematic sequencing errors, single nucleotides in the reads are not always correctly aligned to the reference genome, which can thus lead to wrong conclusions about the allele carried by a sequencing read at the variant site. Thus, allele detection is not a trivial task, especially for single-nucleotide polymorphisms and indels.

**Results:**

To learn the characteristics of sequencing errors, we introduce a method to create an error model in non-variant regions of the genome. This information is later used to distinguish sequencing errors from alternative alleles in variant regions. We show that our method, *k*-merald, improves allele detection accuracy leading to better genotyping performance as compared to the existing WhatsHap implementation using edit-distance-based allele detection, with a decrease of 18% and 24% in error rate for high-coverage Oxford Nanopore and PacBio CLR sequencing reads for sample HG002, respectively. We additionally observed a prominent improvement in genotyping performance for sequencing data with low coverage. For 3× coverage Oxford Nanopore sequencing data, the genotyping error rate reduced from 34% to 31%, corresponding to a 9% decrease.

**Availability and implementation:**

https://github.com/whatshap/whatshap.

## 1 Introduction

Genotyping is a process used for detecting the genotypes of an individual, which further helps in the detection of haplotypes, a task termed as phasing. These processes are widely used in studying the genetic aspects of different diseases and genetic relationships among species. Both genotyping and phasing typically use the alignment between sequencing reads and a reference genome. Thus, prior to genotyping, it is important to determine for each read, whether it carries the reference allele “0” or alternative allele “1” at each of the variant positions it overlaps. Most commonly, short sequencing reads from second-generation sequencing technologies, e.g. Illumina, are used for this purpose because long reads obtained using third-generation sequencing technologies, e.g. Oxford Nanopore technology (ONT) and Pacific BioSciences (PacBio), tend to be more prone to sequencing errors (Zhang *et al.* 2020) unless techniques like circular-consensus sequencing are employed (Wenger *et al.* 2019). However, long reads can be much more informative as they can span longer genomic regions and may cover many variant positions and repetitive regions (Rhoads and Au 2015, Ebler *et al.* 2019).

Over the years, a lot of work has been done to improve basecalling, a process translating raw ONT signal into a DNA sequence. Earlier basecallers employed a two-step process, involving pre-segmentation of raw signals followed by nucleotide label prediction using hidden Markov models (David *et al.* 2017) or recurrent neural networks (Boža *et al.* 2017). Recent years have seen a surge in development of deep learning-based basecallers, dealing directly with the raw signals, hence avoiding error propagation caused by wrong segmentation (Zhang *et al.* 2020). Although state-of-the-art deep learning-based approaches have led to significant improvement in the basecalling accuracy (Wick *et al.* 2019), the error rate for ONT sequencing is still higher than short read sequencing: ONT’s Guppy basecaller achieves basecalling accuracy in a range from 85% to 95% while Illumina Hiseq has basecalling accuracy of around 99.9% (Zhang *et al.* 2020).

Most commonly used read alignment algorithms, such as BWA (Li 2013), do not take sequences of alternative alleles into account for alignment. This results in reference bias (Garrison *et al.* 2018), and pangenomic approaches have been proposed to overcome this problem (Computational Pan-Genomics Consortium 2018, Eizenga *et al.* 2020, Sirén *et al.* 2021, Ebler *et al.* 2022). Despite these developments, alignment to a single linear reference genome remain the standard workflow today. Combined with systematic sequencing errors (Allhoff *et al.* 2013, Wenger *et al.* 2019), this can make alignments at variant sites unreliable to be used for allele detection, thus commonly resulting in sequencing errors being mistaken for an alternative allele. One approach to deal with alignment errors, e.g. employed by WhatsHap (Patterson *et al.* 2015, Martin *et al.* 2016, Porubsky *et al.* 2017), is read re-alignment. The existing implementation of WhatsHap extracts the read sequence from a variant window, 10 bp upstream to 10 bp downstream from the variant position. It then aligns this read sequence to the corresponding reference sequence and to the alternative sequence, produced by interchanging reference with the alternative allele at the variant position. The read is then assigned the allele with lower alignment cost and “unknown” in case of equal scores (Martin *et al.* 2016). The alignment costs are calculated based on edit distance between the sequences. While this technique outperforms the allele detection methods without re-alignment, it does not take systematic sequencing errors into account. Tools like Clair3 (Zheng *et al.* 2022), DeepVariant (Poplin *et al.* 2018), and PEPPER (Shafin *et al.* 2021) perform variant calling and subsequent genotyping of the discovered variants. However, to our knowledge, apart from WhatsHap (Patterson *et al.* 2015, Martin *et al.* 2016, Porubsky *et al.* 2017), there are presently no tools designed specifically for long-read based genotyping of a set of variants given as input.

In this study, we propose a new approach, *k*-merald, for allele detection which is based on the alignment of *k*-mers from reads to *k*-mers from the reference and alternative sequence where alignment costs are based on a learned sequencing error model. *k*-merald, as the name indicates, works in *k*-mer space instead of at the single nucleotide level since *k*-mers help to capture the genomic context in which the systematic errors, specific to a sequencing technology, arise. This approach is based on the idea that genomic regions without any variation can be used to learn the characteristics of sequencing errors. This error model can then be employed to distinguish an allelic variant from a sequencing error at the variant position. Our method first traverses all confident non-variant regions of the genome, recording the sequence and count of the read *k*-mers aligning to each reference *k*-mer (reference-read *k*-mer pairs). These *k*-mer pairs include cases where the two *k*-mers match, indicating an error-free position, or where they mismatch, indicating a sequencing error. The counts of *k*-mer pairs are then used to determine the probability for observing each reference-read *k*-mer pair across the whole genome. We introduce a new approach for global sequence alignment in *k*-mer space. The read, reference, and alternative sequences in each variant window (excluded during the training phase) are split into *k*-mers and the strings of *k*-mers are then aligned. Instead of using a fixed cost value, *k*-mer mismatches are penalized using the learned error model, i.e. *k*-mer mismatches that represent common sequencing errors can be allowed in the alignment at a low cost. The sequencing read is then assigned to the allele with the lowest alignment cost. *k*-merald has been incorporated into the existing WhatsHap implementation and is available as an alternative to the edit-distance-based allele detection.

## 2 Methods

### 2.1 Training the model

**Figure 1.**
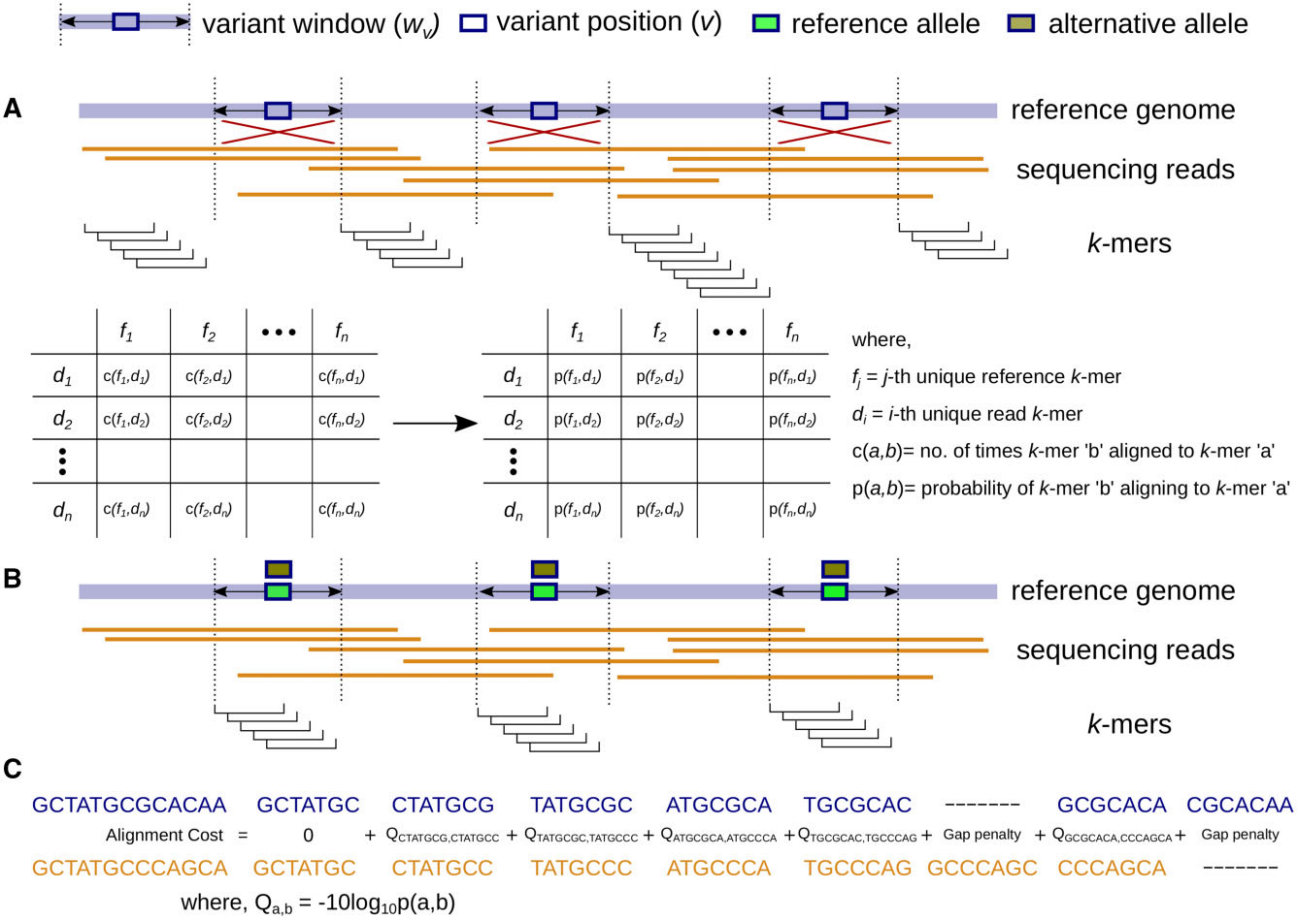
(A) Model training: counts for all the unique reference-read *k*-mer pairs (f,d) in non-variant regions of the genome are recorded. These counts are then used to construct a matrix storing for each unique reference *k*-mer *f*, the probability of seeing each read *k*-mer *d*. (B) Allele detection: a variant window, $w_{v}$, containing the complete variant *v* and a flanking region of a fixed number of *w* base pairs on each side is considered. Both reference and read sequences inside $w_{v}$ are converted into *k*-mers. (C) Alignment: strings of consecutive *k*-mers from each read sequence are aligned individually to the *k*-mer strings obtained from the reference and alternative allelic sequences. A global alignment of the two strings of *k*-mers is done in a similar fashion as global alignment of two base-pair sequences while using phred scores of probabilities, stored during model training, as alignment costs.

As input, we expect a list of candidate variants. In the first step, as shown in Fig. 1A, the sequencing error profile is constructed from non-variant regions of the genome, i.e. regions without candidate variants where the sequencing reads and reference sequence would be identical if sequencing errors were absent. Any changes (e.g. insertions, deletions, substitutions) in the read sequences mapping to these regions can give an indication of the nature of sequencing errors inherent to the sequencing technique that generated the data. Let *F* be the reference sequence excluding all variant windows, where each variant window, $w_{v}$, is defined as an interval containing the complete variant *v* and a flanking region of a fixed number of *w* base pairs on each side. The training data consists of *F* and the set of sequencing reads aligned to it, *D*. Suppose *f* denotes a *k*-mer belonging to *F*, while, *d* denotes a *k*-mer belonging to a sequencing read from *D*. During model training, described in Algorithm 1, *F* is traversed from left to right while maintaining, for each *f*, the count of each mapping *d* using the mapping positions from the input read alignments. For extracting the reference-read *k*-mer combinations (f,d), the read sequence is considered and not the alignment, e.g. if the read *k*-mer AC-GTCT is aligned to the reference *k*-mer ACTGTCT, the respective (f,d) would be (ACTGTCT,ACGTCT*), where * is the nucleotide following ACGTCT in the read sequence. These counts of *k*-mer combinations (f,d) are then aggregated across all occurrences of each reference *k*-mer, to obtain a unique matrix *M* with reference *k*-mers *f* shown in columns (*j*) and read *k*-mers *d* represented in rows (*i*). An entry $M_{ij}$, thus shows the number of times the read *k*-mer $d_{i}$ aligned to the reference *k*-mer $f_{j}$ across the whole length of the reference sequence *F*. Although there are $\;4^{k}$ possible sequence combinations for a *k*-mer of length *k*, many of these combinations are not observed. The (f,d) k-mer combinations that are not observed across the whole length of *F* are each given a pseudocount value ϵ. Instead of representing a presence and absence by “0” and “1,” respectively, a pseudocount value ϵ implies that these *k*-mer combinations can theoretically exist, but have a low probability of occurrence based on our training data. For each reference *k*-mer *f*, we define $K_{f}$ as the set of all *k*-mers *d* aligned to *f*, i.e. the pair (f,d) has an entry larger or equal to 1 in our matrix *M*. The sum of individual counts over all of these pairs is denoted by $t_{f}$. The matrix of counts *M*, is then converted into a matrix *P*, storing the probability of observing each possible reference-read *k*-mer pair (f,d). So, $P_{ij}$ represents the probability of observing a *k*-mer combination $\left(f_{j},d_{i}\right)$ and is calculated as follows:


$$P_{ij}=\frac{M_{ij}}{t_{f_{j}}+(4^{k}-|K_{f_{j}}|)\cdot \epsilon }$$

Table 1 provides an overview of the notations used in this paper. In our implementation, the input data required for this training phase is provided as a VCF file with variant positions, a reference sequence in a FASTA file and a BAM or SAM file containing sequencing reads aligned to the reference sequence. This model training step can be performed using the “learn” module in WhatsHap.

Algorithm 1. Model training

**Input:**

1: The complete reference sequence, *R*

2: Aligned sequencing reads, *B*

3: Variants for which allele detection is to be performed, *V*

**Output:** *M*

4: counter,i⇐ 0

5: v⇐V[counter]

6: **while**i<|R|**do**

7:  **if**i>=v−w & i<=v+w**then**

8:   do nothing

9:  **else if**i>v+w**then**

10:   counter⇐counter+1

11:   v⇐V[counter]

12:  **else**

13:   $k_{R}⇐R[i,i+k-1]$

14:   **for**$b\in \{b^{\prime }\in B|$ alignment of $b^{\prime }$ contains R[i]}**do**

15:    $k_{b}⇐b[j,j+k-1]|b[j]$ aligns to *R*[*i*]

16:    $M[(k_{b},k_{R})]⇐M[(k_{b},k_{R})]+1$

17:   **end for**

18:  **end if**

19:  i⇐i+1

20: **end while**

21: **return** *M*

Problem 1 (Allele detection). Let *V* be a set of all variant positions across the reference genome, let v∈V be a variant position with alleles $a_{1},a_{2},\ldots ,a_{n}$, and let $B_{v}$ be the set of sequencing reads aligned to *v*. Determine $a_{v_{b}}$ for each sequencing read b∈B, where $a_{v_{b}}$ denotes the allele carried by *b* at position *v*.

**Table 1.** Overview of used notations.

Notation	Definition
*R*	The complete reference sequence
*B*	Sequencing reads aligned to *R*
*V*	A set of variants for which allele detection is to be performed
$w_{v}$	A window of fixed number of *w* base pairs on each side of v∈V
*F*	*R* excluding $w_{v}$ for all v∈V
*D*	*B* excluding parts of read sequences mapping to a $w_{v}$ for all v∈V
*f*	A *k*-mer belonging to *F*
*d*	A *k*-mer belonging to *D*
*M*	A matrix recording the number of occurrences of each reference-read *k*-mer pair, (f,d)
*P*	A matrix recording the probability of occurrence of each reference-read *k*-mer pair, (f,d)

Definition 1 (Minimum cost allele). Let $b_{v}$ be the read sequence segment aligned to a variant window $w_{v}$, i.e. the read *b* sequence from the window around variant *v* and let $Q=[q_{1},\ldots .,q_{n}]$ be the set of all possible allele sequences belonging to $w_{v}$, i.e. $q_{1}$ corresponds to $w_{v}$ sequence with reference allele at *v* and $q_{2},\ldots ,q_{n}$ to the sequences with alternative alleles at *v*. If d(x,y) denotes the alignment cost for two sequences *x* and *y*, then

$$a_{v_{b}}=\underset{i\in \{1,\ldots ,n\}}{\text{arg min}}d(b,q_{i})$$

where, $a_{v_{b}}$ denotes the allele carried by *b* at position *v*.

### 2.2 Alignment algorithm

Our next goal is to use the probability matrix *P*, which represents our model of sequencing errors, to define an alignment cost d(x,y) and, based on this, to determine the minimum cost allele (Definition 1). Therefore, for a given variant position, we seek to determine whether an observed sequencing read is more likely to have originated from the reference allele or from one of the alternative alleles. In this phase, we only deal with variant windows, i.e. the regions that were not considered in the model training phase. The read sequences from each $w_{v}$ are mapped to both the reference and alternative sequence of the respective $w_{v}$, as shown in Fig. 1B. The reference sequence for each $w_{v}$ is extracted directly from the reference genome, while the alternative sequence is obtained by replacing the reference allele with the alternative allele at the variant position. For alignment, we developed a modified version of the Needleman–Wunsch algorithm (Needleman and Wunsch 1970). This modified algorithm, described formally in Algorithm 2, performs *k*-mer-based comparisons (Fig. 1C) instead of the conventional single-character based sequence comparison. Each sequence is first converted into a string of consecutive *k*-mers and the resulting strings are then aligned by comparing respective *k*-mers. The algorithm uses “phred-scaled” probability scores (−10⋅ log(probability)) for alignment cost calculation, where probability values are obtained from the matrix *P* learned from the training phase. This cost model is used to penalize mismatches when the reference *k*-mer and the read *k*-mer are not identical. The mismatching *k*-mer pairs frequently observed across the non-variant positions, *F*, due to systematic sequencing errors, hence having a high probability in matrix *P*, get a lower penalty as compared to those seen occasionally due to sporadic sequencing errors. For gaps, the probability value can be specified by the user as a parameter, which we set to $10^{-4}$ in this study, i.e. a cost value of “40.”

In summary, by design, the algorithm ensures that a read carrying a sequencing error aligns to the reference with a cost lower than to the alternative allele, thus minimizing the risk of a sequencing error being mistaken for a variant allele. The read is assigned the allele resulting in lowest alignment cost. However, equal alignment costs indicate that the algorithm was unable to make an allele detection based on the alignment. In case of multiallelic variants, the alignment is performed using each alternative sequence. *k*-merald has been implemented inside WhatsHap and can be used as an alternative approach for allele detection in (i) haplotagging, the process to label each read with a haplotype of origin, (ii) genotyping, and (iii) phasing.

Algorithm 2. *k*-mer alignment

**Input:**

1: List of *k*-mers from the target sequence, $S_{1}$

2: List of *k*-mers from the query sequence, $S_{2}$

3: Gap penalty, $C_{\text{gap}}$

4: *P*

**Output:** Optimal cost for aligning $S_{1}$ to $S_{2}$

5: **for**i←0 to $\mathrm{length}(S_{1})$**do**

6:  $DP[i,0]⇐C_{\text{gap}}*i$

7: **end for**

8: **for**j←0 to $\mathrm{length}(S_{2})$**do**

9:  $DP[0,j]⇐C_{\text{gap}}*j$

10: **end for**

11: **for**i←1 to $\mathrm{length}(S_{1})$**do**

12:  **for**j←1 to $\mathrm{length}(S_{2})$**do**

13:   $C_{\text{match}}⇐DP[i-1][j-1]+P_{S_{1[i-1]},S_{2[j-1]}}$

14:   $C_{\text{delete}}⇐DP[i-1][j]+C_{\text{gap}}$

15:   $C_{\text{insert}}⇐DP[i][j-1]+C_{\text{gap}}$

16:   $DP[i][j]⇐\min(C_{\text{match}},C_{\text{delete}},C_{\text{insert}})$

17:  **end for**

18: **end for**

19: **return**$DP[\mathrm{length}(S_{1}),\mathrm{length}(S_{2})]$

## 3 Results

### 3.1 Sequencing error profiles

**Figure 2.**
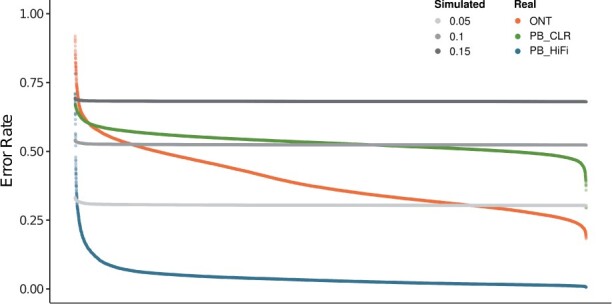
Distribution of 7-mer error rates observed for simulated and real long-read datasets. The simulated long reads have a uniform base-line error rate distribution with an error rate of 0.05, 0.1 and 0.15, each using a read length of 20 kb and 35× mean coverage across available positions. The rate of mutations was set to 0.0010, of which 10% are indels. The real dataset includes sequencing reads from ONT, PacBio CLR, and PacBio HiFi for sample HG002. The *x*-axis represents the unique *k*-mers belonging to the GRCh38 reference genome.

**Figure 3.**
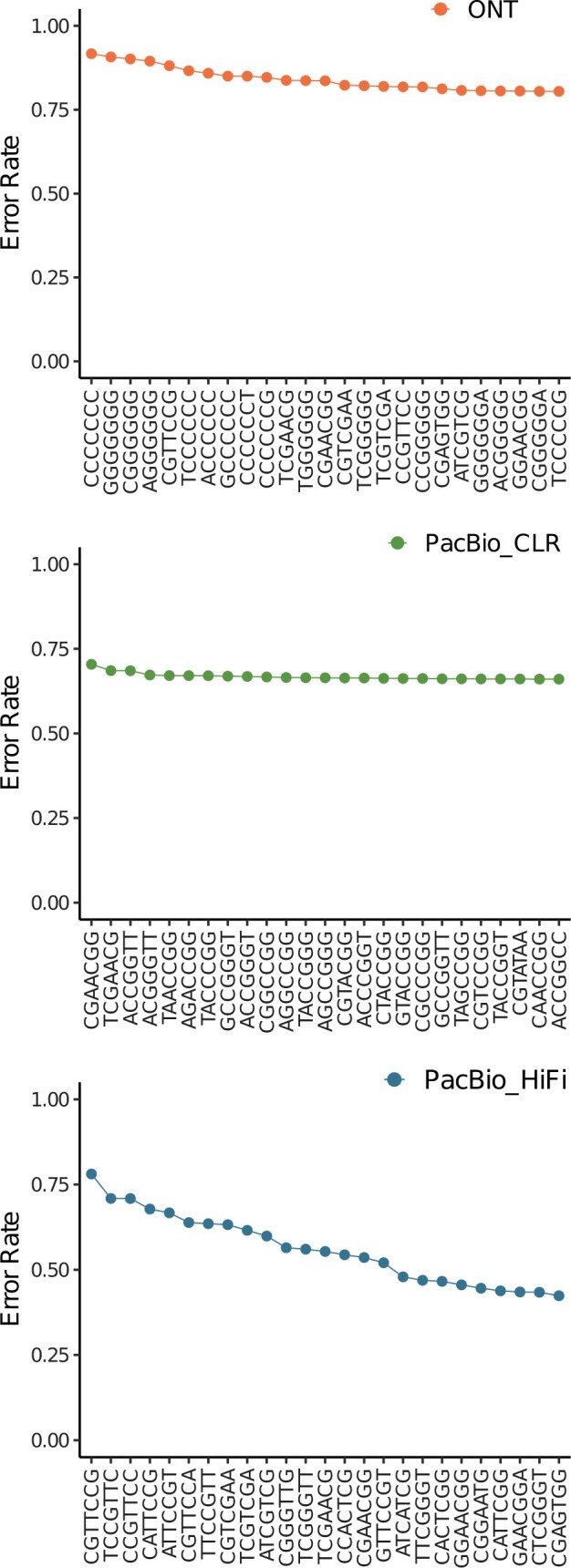
Error rates for the 25 top most erroneous 7-mers belonging to the GRCh38 reference genome for sequencing reads from ONT, PacBio CLR, and PacBio HiFi, individually.

We first visualized the sequencing error profiles for Oxford Nanopore, PacBio CLR, and PacBio HiFi, respectively. These profiles were generated using sequencing reads from sample HG002 aligned to human reference genome GRCh38. For comparison, we generated simulated long-read data with uniform error distribution with an error rate of 0.05, 0.1, and 0.15, each with an average read length of 20 kb and 35× mean coverage across available positions. The rate of mutations was set to 0.0010, of which 10% are indels. The aligned simulated reads and simulated variants were used for generation of the error profiles as described in Algorithm 1. That is, this process also captures any alignment artifacts that might be present. Figure 2 shows the error profiles generated by setting *k *=* *7 and *w *=* *25. The error rate for each reference *k*-mer represents the sum of probabilities of observing each *k*-mer pair (f,d) such that d≠f. Figure 2 shows that in contrast to the error rate pattern observed for data with uniform base-line error rate, the error rate distribution differs across the sequencing technologies and is non-uniform for each of them. A closer look at the 25 most erroneous *k*-mers for ONT, PacBio CLR, and PacBio HiFi, each, reveals that the nature of erroneous *k*-mers also differs across the sequencing technologies (Fig. 3). The erroneous *k*-mers from PacBio CLR seem to be more GC-rich while ONT erroneous *k*-mers appear to be AT rich. The fact that these error distributions are not uniform and distinct from one another supports our hypothesis that considering technology-specific error profiles can help improve allele detection accuracy.

GIAB variant callsets come with a designation of high confidence regions in which the callsets can be considered complete. However, for the remainder of the genome, they are less complete. To assess the impact of missing variant positions on the error profiles, we evaluated the genotyping performance across error models learned using multiple variant callsets. Each of these callsets contained only a percentage of variants, ranging from 1% to 95%, from the full GIAB benchmark callset. We observed that the genotyping error rates as shown in Supplementary Fig. S1 remain almost unaffected even after excluding a large fraction of variant positions, hence proving the robustness of our training method.

### 3.2 Comparison to WhatsHap genotyping

Correct allele detection from individual reads plays a pivotal role in genotyping. So, we compared our genotyping results with those obtained using WhatsHap’s original implementation. We based our evaluation on two Genome in a Bottle (GIAB) samples, HG001 (NA12878) and HG002 (NA24385). We used whatshap genotype for genotyping the GIAB v4.2.1 high confidence benchmark callsets (Wagner *et al.* 2022). We performed comparisons using various coverages of ONT ultra-long, PacBio CLR and PacBio HiFi sequencing reads. To evaluate genotyping performance, we calculated genotype concordance, i.e. the percentage of variants genotyped correctly. Additionally, we used RTG Tools “vcfeval” (Cleary *et al.* 2015) to calculate precision, sensitivity, and *F*_1_ score for the predicted genotypes. Finally, we used GIAB v3.0 stratifications to compare the genotyping performance in difficult-to-map and low-complexity regions of the genome.

**Figure 4.**
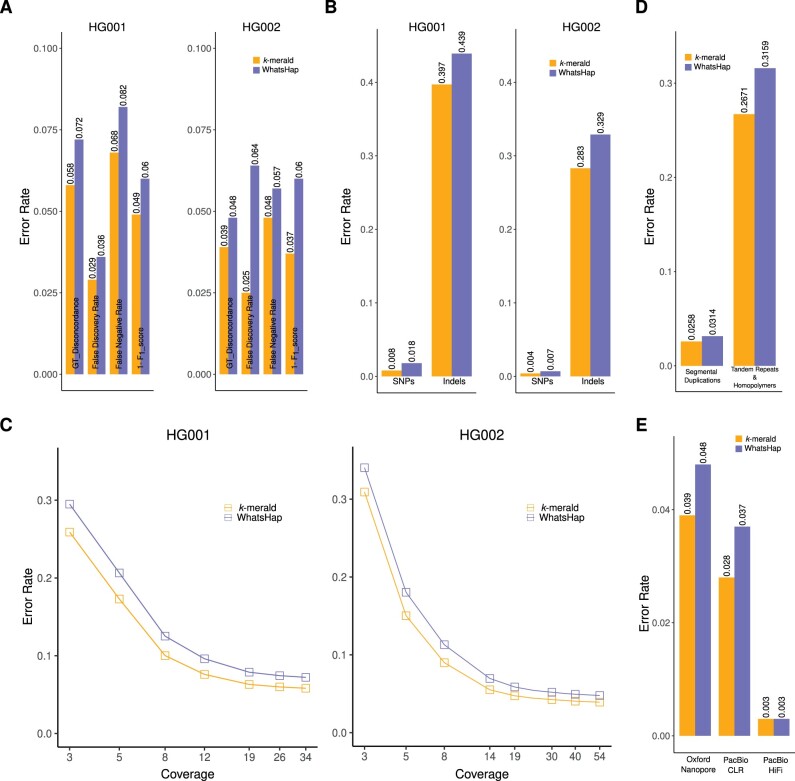
(A) Genotyping performance comparison between WhatsHap with conventional edit-distance-based allele detection and *k*-merald using ONT sequencing reads for sample HG001 and HG002. (B) Genotyping performance comparison for SNPs and indels, individually, using ONT sequencing reads. (C) Genotyping performance comparison across multiple coverages of ONT sequencing reads. (D) Genotyping performance comparison across multiple genome stratifications using ONT sequencing reads for sample HG002. (E) Genotyping performance comparison, individually for ONT, PacBio CLR, and PacBio HiFi data for sample HG002.

We first evaluated the genotyping performance using ONT sequencing reads for GIAB samples HG002 and HG001. We used a *k*-mer value of *k *=* *7, variant window *w *=* *25 and ϵ = 0.15 for the genotyping results presented in this study. A comparison of genotyping error rates across multiple values of *k* is shown in Supplementary Fig. S2. Considering single-nucleotide polymorphisms (SNPs) and indels together, we observed that genotyping using *k*-merald for allele detection shows an improved performance in comparison to WhatsHap’s genotyping results based on the conventional edit-distance-based allele detection approach. For 54× HG002 ONT sequencing reads, the genotype concordance improved from 95.22% to 96.08%, indicating a 18.12% decrease in error rate (Fig. 4). Precision, sensitivity, and *F*_1_ score values also depict this improvement (Fig. 4A). To assess the robustness, we also evaluated the genotyping performance for sample HG001, using the error profiles trained using ONT sequencing data for HG002. A similar trend was observed for the 34× HG001 ONT sequencing reads, with genotype concordance improving from 92.78% to 94.18% indicating a 19.41% decrease in error rate (Fig. 4A). This consistent improvement in genotyping performance seen while using different samples for training and testing confirms that the characteristics of error profiles captured by *k*-merald are technology specific, instead of being sample specific. Thus, an error profile generated using only one sample can be readily used for genotyping multiple samples with sequencing data generated from the same source.

Furthermore, we evaluated the genotyping performance individually for SNPs and indels. For HG001, we observed 55.07% decrease in error rate for SNPs and 9.52% for indels. For HG002, the percentage decrease was 47.42% and 13.94%, for SNPs and indels, respectively (Fig. 4B).

We hypothesized that using our sequencing error profiles would also improve the process of estimating genotype quality values, particularly for indels. That is, the method is better able to assess the reliability of genotypes and to express it as a genotype quality provided along with the genotypes, which is potentially beneficial for downstream applications. To evaluate this, we compared the genotype quality between *k*-merald and edit-distance-based genotypes for GIAB v4.2.1 whole-genome high confidence indels, genotyped using 54× ONT data for sample HG002. We observed that the correct *k*-merald genotypes tend to be of higher genotype quality as compared to the correct genotypes obtained using WhatsHap’s genotyping using edit-distance-based allele detection. In total, 67% of the correct *k*-merald genotypes exhibited a genotype quality of at least 200, while this percentage was 60% for WhatsHap genotypes. For all genotypes with a quality of at least 200, the percentage of correct genotypes was 89% for *k*-merald while 85% for WhatsHap (Supplementary Fig. S3).

We reasoned that the negative impact of sequencing errors on allele detection might become even more prominent at low coverage, and therefore evaluated the genotype performance across multiple coverages of sequencing reads. For HG002, we downsampled the Oxford Nanopore data to coverages ranging from 3× to 54×. For HG001, we downsampled the Oxford Nanopore data to coverages ranging from 3× to 34×. For both these samples, we observed, in line with our hypothesis, that although our new approach outperforms the conventional allele detection algorithm at all coverages, the absolute difference becomes more pronounced at lower coverages (Fig. 4C).

Additionally, we compared the genotyping performance in low mappability segmental duplications as well as low complexity regions like tandem repeats (dinucleotide, trinucleotide and quadnucleotide STRs, and simple repeats) and homoploymers (perfect homopolymers >6 bp and imperfect homopolymers >10 bp). We observed that across all these regions, *k*-merald gives better genotyping performance than the conventional edit-distance-based genotyping with 15% decrease in error rate for tandem repeats and homopolymers and 18% for segmental duplications (Fig. 4D).

Finally, to evaluate performance across different sequencing platforms, we evaluated the results obtained by using PacBio CLR and PacBio HiFi sequencing reads. For 20× HG002 PacBio CLR sequencing reads, the genotype concordance improved from 96.32% to 97.24% indicating a 24% decrease in error rate. For 35× HG002 PacBio HiFi sequencing reads, we observed both approaches to show very similar genotyping performance (Table 2 and Fig. 4E). This supports the hypothesis that our method provides a particular advantage for more error-prone sequencing reads.

**Table 2.** Genotyping performance for HG002.

	GT concordance (%)	Precision (%)	Sensitivity (%)	*F*_1_ score (%)
ONT-UL				
WhatsHap	95.22	93.57	94.34	93.95
*k*-merald	96.08	97.46	95.19	96.31
PacBio CLR				
WhatsHap	96.32	97.65	93.97	95.78
*k*-merald	97.24	97.89	94.90	96.37
PacBio HiFi				
WhatsHap	99.70	99.75	98.77	99.26
*k*-merald	99.67	99.78	98.74	99.26

For 54× Oxford Nanopore reads, generating genome-wide error profile took about 145 CPU hours collectively. Whole-genome genotyping collectively took about 29 single-core CPU hours using whatshap genotype with conventional edit-distance-based allele detection, while about 139 single-core CPU hours using whatshap genotype with *k*-merald. We attribute the increased running time to the more involved bookkeeping for working with *k*-mers in Algorithm 2 compared to the single-nucleotide sequence alignment. However, we note that the steps were performed in parallel in a chromosome-wise manner. Given the running time of read alignment that happens before genotyping, we do not consider this increased runtime to be the main bottleneck in processing a long read dataset.

### 3.3 Comparison to PEPPER

**Figure 5.**
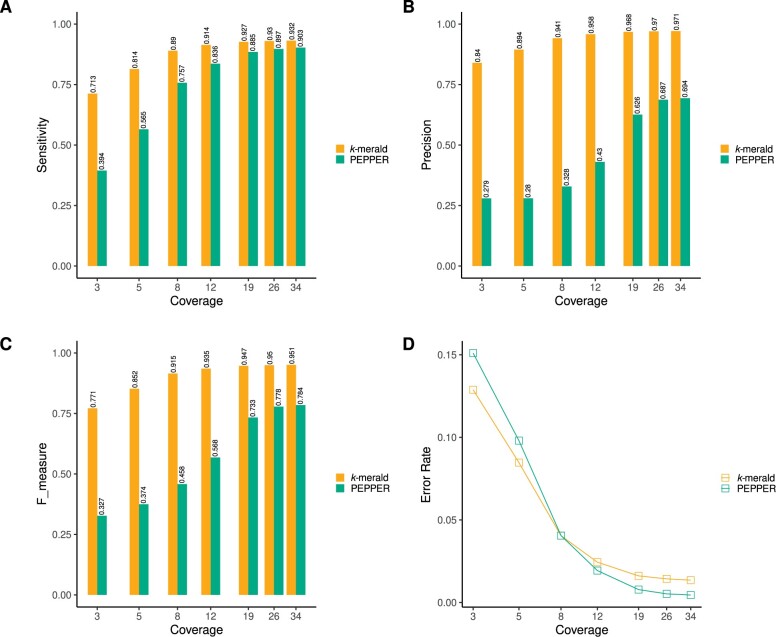
(A) Comparison of genotyping precision between *k*-merald and PEPPER for sample HG001, across multiple coverages of ONT sequencing reads, using high confidence GIAB v4.2.1 genotypes as ground truth. For *k*-merald-based genotyping, we used error profiles generated for HG002. (B) Comparison of genotyping sensitivity between *k*-merald and PEPPER for sample HG001, across multiple coverages of ONT sequencing reads, using high confidence GIAB v4.2.1 genotypes as ground truth. (C) F_measure comparison between *k*-merald and PEPPER for sample HG001, across multiple coverages of ONT sequencing reads, using high confidence GIAB v4.2.1 genotypes as ground truth. (D) Error rate comparison between *k*-merald and PEPPER for sample HG001, across multiple coverages of ONT sequencing reads. The comparison was restricted to variants common between *k*-merald and PEPPER callsets and high confidence GIAB v4.2.1 genotypes were used as ground truth.

We aimed to compare our approach to the state-of-the-art tool PEPPER (Shafin *et al.* 2021), which detects candidate variants, genotypes, and phases them in an integrated workflow. Comparing a genotyper’s performance to such an integrated variant caller is not a straight-forward process. To avoid a skewed comparison, we performed this comparison in two ways. Firstly, we computed precision, recall, and *F*_1_ score for all the variants called/genotyped by each method in their respective default mode. That is, our method is provided with the set of all variants to be genotyped as input, while PEPPER runs both discovery and genotyping. We performed this comparison using multiple coverages of Oxford Nanopore reads for sample HG001, while using the error profiles for HG002. For all these measures, we observed that our method performed better as compared to PEPPER at all coverages (Fig. 5A–C). However, it should be noted that PEPPER had to perform the additional step of variant discovery before genotyping. Therefore, this evaluation method could potentially favor the genotyper. To address this, we additionally computed genotype concordance only for the variants common between GIAB v4.2.1 callset and the PEPPER callset. Even though this method of comparison favors PEPPER, as we restrict our evaluation only to the variants that could be called by the variant caller, we observed that our method still gives lower error rate as compared to PEPPER for low coverage data (Fig. 5D).

## 4 Discussion

Correct detection of alleles carried by sequencing reads is vital for variant genotyping and haplotype phasing (Glusman *et al.* 2014). In comparison to short reads, long reads span larger regions, hence providing more information. However, sequencing errors generated by long-read sequencing technologies pose a challenge for allele detection. The sequencing error profiles vary across multiple sequencing technologies such as ONT, PacBio CLR, and PacBio HiFi. That includes different error distributions as well as different characteristics of sequencing errors (Fig. 2). The conventional allele detection methods are mostly based on edit distance, which penalizes all sequence mismatches equally. We hypothesized that instead of fixed costs, using technology-specific sequencing error profiles for determining alignment costs can provide more insights to distinguish a variant allele from a sequencing error, hence improving the allele detection accuracy. To address this, we proposed a method that generates technology-specific *k*-mer-based error profiles by traversing aligned sequencing reads in the non-variant regions of the genome. We also developed a *k*-mer-based alternative to global sequence alignment that uses the error profiles for alignment cost calculation. This method, instead of aligning the sequences of base pairs, aligns strings of consecutive *k*-mers generated from the respective sequences.

We observed that WhatsHap genotyping using *k*-merald results in better genotyping performance as compared to the existing WhatsHap implementation, which detects alleles using edit-distance-based sequence alignment. We observed 18% and 24% decrease in genotyping error rate for 54× ONT and 20× PacBio CLR sequencing reads, respectively. The genotyping performance, however, was similar for PacBio HiFi sequencing data potentially because of their lower error rate as compared to ONT and PacBio CLR. While evaluating the genotyping performance individually, we observed a 47% decrease in error rate for SNPs while 14% for indels, for sample HG002. A comparison of genotyping performance across multiple coverages of ONT data revealed that the improvement in genotyping performance shown by our new approach becomes even more prominent at low coverages.

At present, ONT is the most cost-effective long-read sequencing platform in terms of costs per sequenced base pair. But this comes at the disadvantage of increased and more systematic error profiles. Our method provides substantial improvements in allele detection in order to push genotyping performance to its limits. Of note, the use of error models trained for a given sequencing dataset provides a way to take technology-specific differences into account when computing genotype likelihoods, hence allowing us to quantify uncertainty in a more informed way. This is reflected in our results showing that variants genotyped with high genotype quality (GQ) above 200 are more strongly enriched for correct genotypes when using *k*-merald.

Our training procedure exploits the similarity of a sequenced sample and the reference genome by using variant-free regions for training. In this way, our model can be readily retrained even on a single dataset, which potentially allows it to adapt to subtle differences such as version of the sequencing chemistry and other batch effects. Because the learning procedure is technology agnostic, we anticipate that our method can readily be applied to future long read data types.

## Supplementary Material

vbad149_Supplementary_Data

## Data Availability

Links to all the sequencing data used in this study can be found in the Data availability section in the Supplementary Data. The source code is available at https://github.com/whatshap/whatshap.
