# Supplementary material for: Allele detection using k-mer-based sequencing error profiles
Source: Bioinform Adv. 2023 Oct 20;3(1):vbad149. doi: 10.1093/bioadv/vbad149 (PMC10625474; doi:10.1093/bioadv/vbad149)
Supplement: vbad149_Supplementary_Data [file vbad149_supplementary_data.pdf]

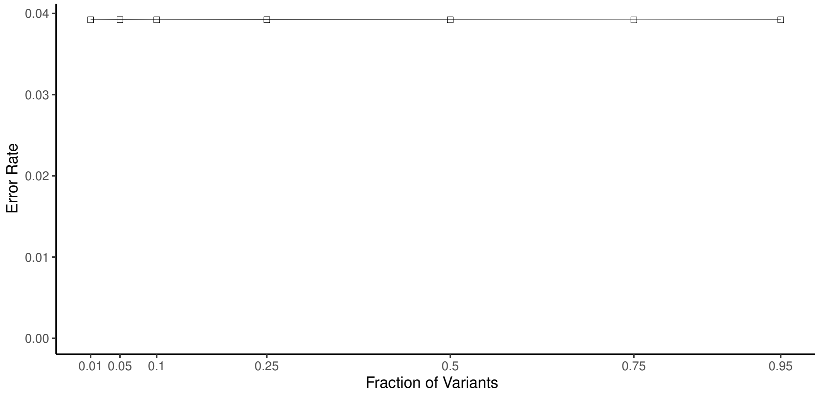

Figure S1: Assessment of the robustness of k-merald by comparing the genotyping performance across error models learned using multiple variant callsets. Each of these callsets contained only a percentage of variants, ranging from 1% to 95%, from the original GIAB v4.2.1 benchmark callset. x-axis represents the fraction of variants used for the training phase, while y-axis represents the corresponding genotyping error rate.

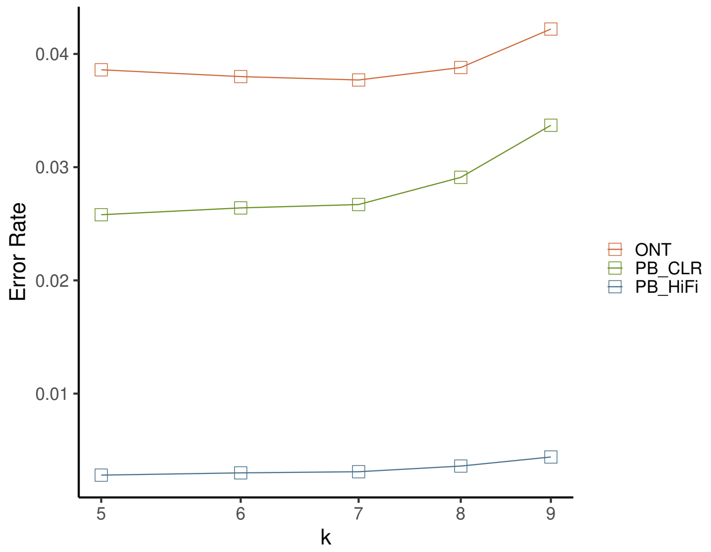

Figure S2: Error rates observed across different values of k in chr22 for Oxford Nanopore, PacBio HiFi and PacBio CLR.

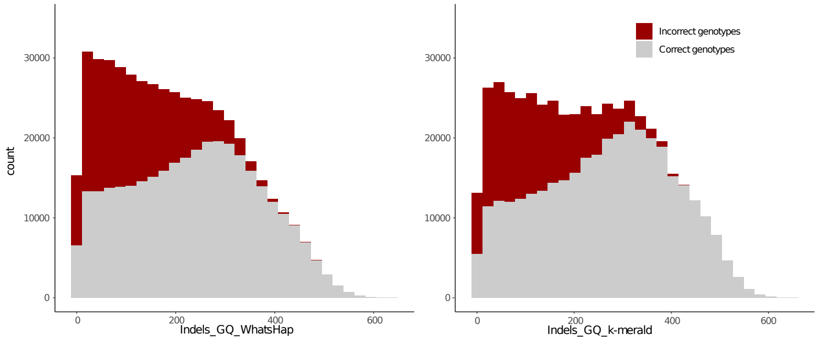

Figure S3: A comparison of whole genome indels genotype quality between k-merald and Whatshap for sample HG002 using ONT sequencing data.

1 Data Availability

1.1 PacBio CLR

HG002: https://s3-us-west-2.amazonaws.com/human-pangenomics/NHGRI_UCSC_panel/HG002/hpp_HG002_NA24385_son_v1/PacBio_CLR/PB_HG002-CLR-SvDetection/m64070_190824_163708.subreads.bam
pbmm2 was used for alignment to GRCh38

```
pbmm2 align GRCh38.fa m64070_190824_163708.subreads.bam
↪  alignments.bam --sort --median-filter --sample HG002
```

1.2 PacBio Hifi

HG002: https://s3-us-west-2.amazonaws.com/human-pangenomics/working/HPRC_PLUS/HG002/analysis/aligned_reads/hifi/GRCh38/HG002_aligned_GRCh38_winnowmap.sorted.bam

1.3 Oxford Nanopore

HG002: https://ftp-trace.ncbi.nlm.nih.gov/giab/ftp/data/AshkenazimTrio/HG002_NA24385_son/Ultralong_OxfordNanopore/guppy-V3.4.5/HG002_GRCh38_ONT-UL_GIAB_20200204.bam
HG001: https://ftp-trace.ncbi.nlm.nih.gov/giab/ftp/data/NA12878/Ultralong_OxfordNanopore/NA12878-minion-ul_GRCh38.bam

1.4 Variant callsets

HG002: https://ftp-trace.ncbi.nlm.nih.gov/giab/ftp/release/AshkenazimTrio/HG002_NA24385_son/latest/GRCh38/HG002_GRCh38_1_22_v4.2.1_benchmark.vcf.gz
HG001: https://ftp-trace.ncbi.nlm.nih.gov/giab/ftp/release/NA12878_HG001/latest/GRCh38/HG001_GRCh38_1_22_v4.2.1_benchmark.bed

1.5 Genome stratifications

https://ftp-trace.ncbi.nlm.nih.gov/giab/ftp/release/genome-stratifications/v3.0/GRCh38/
